# Prostaglandin E_2_ (PGE_2_) in tissue regeneration: Its role and therapeutic strategies

**DOI:** 10.17179/excli2025-9025

**Published:** 2025-11-28

**Authors:** Wenlong Wang, Kai Pan, Jun Yang, Zongjin Li

**Affiliations:** 1 Yantai Yuhuangding Hospital affiliated to Qingdao University, Yantai, China; 2 Qingdao University, Qingdao, China; 3 Nankai University School of Medicine, Tianjin, China

**Keywords:** Prostaglandin E2 (PGE2), E-type prostanoid (EP) receptor, tissue regeneration, stem cells, therapeutic strategy, delivery

## Abstract

Prostaglandin E₂ (PGE₂), which is traditionally recognized as a pro-inflammatory mediator target, is now recognized for its role in tissue regeneration. PGE₂ drives stem cell proliferation, M2 macrophage polarization, angiogenesis, and extracellular matrix (ECM) remodeling via E-type prostanoid (EP) receptor signaling, promoting repair in the skin, muscle, bone, heart, liver, kidney, and intestine. Despite these promising effects, the clinical translation of PGE₂ has been hindered by challenges such as a short half-life due to rapid degradation by 15-hydroxyprostaglandin dehydrogenase (15-PGDH), limited EP receptor subtype specificity, or oncogenic risks in certain contexts. This review explores the regenerative mechanisms of PGE₂, its tissue-specific roles, and innovative strategies to optimize therapeutic efficacy while minimizing adverse effects in regenerative medicine.

See also the graphical abstract(Fig. 1).

## Introduction

Tissue regeneration is a coordinated physiological process essential for restoring structural and functional integrity after injury (Zhao et al., 2021[102]). This process relies on immune modulation, stem cell activation, and extracellular matrix (ECM) remodeling, and its dysregulation underlies conditions such as chronic ulcers, fractures, and organ dysfunction (Cheng et al., 2021[14]). Among the key molecular mediators, PGE₂ has emerged as a central regulator of repair across multiple organ systems, including the skin, heart, liver, kidney, gut, bone, skeletal muscle, and hematopoietic system (Chen et al., 2021[12], 2024[13], Cheng et al., 2021[14], Huang et al., 2022[39]). PGE₂ promotes inflammatory resolution and enhances regenerative responses; however, its rapid degradation by dehydrogenases, receptor subtype-dependent effects, and inhibition by nonsteroidal anti-inflammatory drugs limit its therapeutic translation (Geusens et al., 2013[26], Xiao et al., 2004[90]). To overcome these barriers, novel strategies have been developed, including pharmacological augmentation of endogenous PGE₂ with dehydrogenase inhibitors (Palla et al., 2021[70]), biomaterial-based systems for targeted and sustained delivery (Chen et al., 2021[12]), and mesenchymal stromal cell (MSC) priming with PGE₂ to increase reparative potency (Du et al., 2025[20]). Together, these approaches highlight the potential of PGE₂-centered interventions to maximize regenerative efficacy while minimizing adverse effects, underscoring its promise as a therapeutic axis in regenerative medicine.

## Biochemical Processes and Physiological Roles of PGE₂

### Biochemical Processes and Physiological Roles of PGE_2_

**Figure 2:**
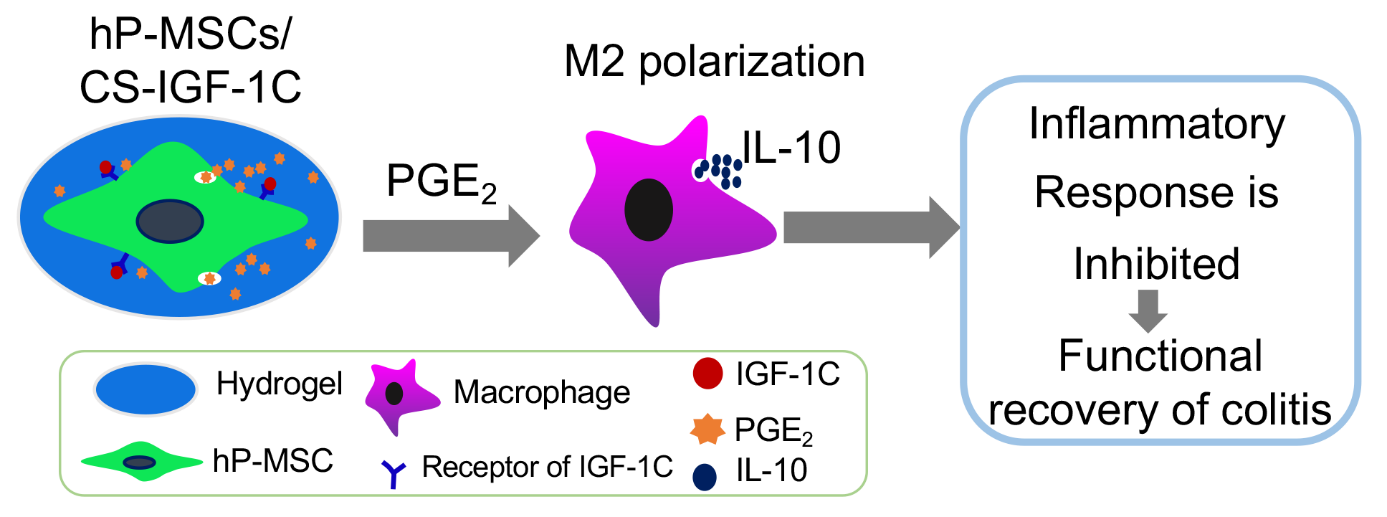
A strategy to promote PGE₂ secretion from MSCs by co-transplanting MSCs with bioactive compounds, the CS-IGF-1C hydrogel, can amplify the therapeutic effects of MSCs. The composite system operates by protecting the delivered MSCs and augmenting their paracrine release of PGE₂. This promotes a pro-resolving immune microenvironment via M2 macrophage polarization, thereby mitigating inflammation and stimulating regenerative processes that lead to colon recovery. CS-IGF-1C, chitosan (CS)-based injectable hydrogel with immobilized IGF-1 C domain peptide; hP-MSCs, human placental mesenchymal stromal cells. Reproduced with permission from Ref (Cao et al., 2020).

PGE₂ is a critical mediator not only of pain and inflammation but also of a wide array of physiological functions, such as the regulation of vascular tone, maintenance of gastrointestinal integrity, renal homeostasis, hematopoiesis, and immune responses. These pleiotropic actions are derived from tightly controlled biosynthesis, rapid turnover, and receptor-specific signaling (Cheng et al., 2021[14]). MSCs are widely studied for their therapeutic potential in regenerative medicine, and PGE_2_ is a key paracrine factor secreted by MSCs that contributes to their immunomodulatory effects (Cao et al., 2020[8]). Co-transplantation MSCs with bioactive compounds can increase PGE2 secretion, which can amplify these benefits (Figure 2(Fig. 2); Reference in Figure 2: Cao et al., 2020[8]). Increasing evidence indicates that these properties confer a central role for PGE₂ in tissue protection and regeneration. Defining its biochemical synthesis, signaling pathways, and cellular targets is essential for developing PGE₂-based regenerative therapies.

### Synthesis and signaling pathways of PGE_2_

PGE₂ is synthesized from arachidonic acid (AA), which is released from membrane phospholipids by phospholipase A₂ (PLA₂). AA is then converted to prostaglandin H₂ (PGH₂) by cyclooxygenases (COXs), a family of enzymes that catalyze the rate-limiting step in prostanoid biosynthesis (Harris et al., 2002[31]). COX-1 is constitutively expressed and maintains physiological homeostasis, whereas COX-2 is inducible and upregulated in response to inflammatory stimuli or tissue injury. PGH₂ is subsequently isomerized to PGE₂ by prostaglandin E synthases (PGES), including inducible mPGES-1, which couples with COX-2 and constitutively expresses cPGES and mPGES-2 (Park et al., 2006[71]). This pathway is inhibited by nonsteroidal anti-inflammatory drugs (NSAIDs), which reduce PGE₂ production.

PGE₂ levels are further regulated by cellular transport and metabolism. Efflux is mediated by multidrug resistance protein 4 (MRP4/ABCC4), whereas uptake occurs via the prostaglandin transporter (PGT) SLCO2A1 (Reid et al., 2003[74]). The main catabolic enzyme, 15-hydroxyprostaglandin dehydrogenase (15-PGDH), converts PGE₂ to inactive 15-keto-PGE₂ (Guda et al., 2014[28], Nakanishi et al., 2021[63]). PGE₂ signals through four G protein-coupled E-type prostanoid (EP) receptors. EP1 elevates intracellular Ca²⁺ and activates protein kinase C (PKC), EP3 inhibits adenylate cyclase and reduces cAMP, and EP2/EP4 activate adenylate cyclase, increasing cAMP and activating protein kinase A (PKA). These pathways interact with the MAPK/ERK, PI3K/Akt, NF-κB, and TGF-β/SMAD signaling pathways to coordinate context-dependent cellular responses.

### Multifaceted effects of PGE_2_ on cellular function

PGE_2_ mediates tissue repair primarily through EP2/EP4cAMPPKA signaling, promoting the proliferation and activation of tissue-resident stem and progenitor cells, as well as epithelial cells, to restore structural integrity (Konger et al., 2005[52], Wei et al., 2019[89]). These include cardiac stem cells, Lgr5⁺ intestinal stem cells, hematopoietic stem cells (HSCs) and MSCs (Cheng et al., 2021[14], Han et al., 2020[30]). It also accelerates the proliferation of epithelial cells for barrier restoration (Liu et al., 2023[59]). Concurrently, it orchestrates spatiotemporal recruitment of repair cells, directs immunomodulatory macrophage polarization toward pro-regenerative M2 phenotypes, and suppresses fibrosis via TGF-β1/SMAD3 pathway inhibition to collectively establish a regenerative microenvironment while exhibiting context-dependent differentiation outcomes.

PGE₂ functions as a potent chemoattractant, orchestrating the cellular migration essential for wound repair. It stimulates the recruitment of fibroblasts and endothelial cells to cutaneous wounds, which is primarily mediated by the EP4 receptor, and facilitates the mobilization of wound-associated epithelial cells (WAEs) to achieve re-epithelialization of gastrointestinal mucosal defects (Jackstadt and Sansom, 2017[43]). Furthermore, PGE₂ enhances the homing capacity of exogenously administered therapeutic cells, such as MSCs, to sites of injury through the modulation of chemokine signaling networks (Cheng et al., 2022[15]). PGE₂ critically regulates cellular differentiation and fate. PGE₂ induces myogenic differentiation in muscle stem cells and osteogenic differentiation in MSCs, primarily through the EP4 receptor and the cAMP‒PKA pathway (Ho et al., 2017[35], Lin et al., 2017[57], Wang et al., 2025[88]). It further supports the reconstruction of gastric mucosal glands and promotes goblet cell differentiation (Hatazawa et al., 2007[32]).

Crucially, PGE₂ serves as a potent immunomodulator, driving macrophage polarization from the pro-inflammatory M1 phenotype to the reparative M2 phenotype in an EP2/EP4-dependent manner. These M2 macrophages secrete anti-inflammatory mediators, suppress pro-inflammatory cytokines, and establish a regenerative microenvironment (Pajarinen et al., 2019[69]). PGE₂ also exerts significant anti-fibrotic effects by inhibiting the TGF-β1/SMAD3 signaling pathway, downregulating α-smooth muscle actin (α-SMA) and matrix metalloproteinase-2 (MMP2), and suppressing myofibroblast differentiation and collagen deposition (Zhang et al., 2025[96]). Notably, PGE₂-mediated differentiation outcomes are context dependent, as observed in gut injury, where PGE₂ promotes transient WAE generation rather than standard epithelial differentiation processes (Jackstadt and Sansom, 2017[43], Miyoshi et al., 2016[61]).

## PGE₂ as an Orchestrator of Tissue Repair and Regeneration

**Figure 3:**
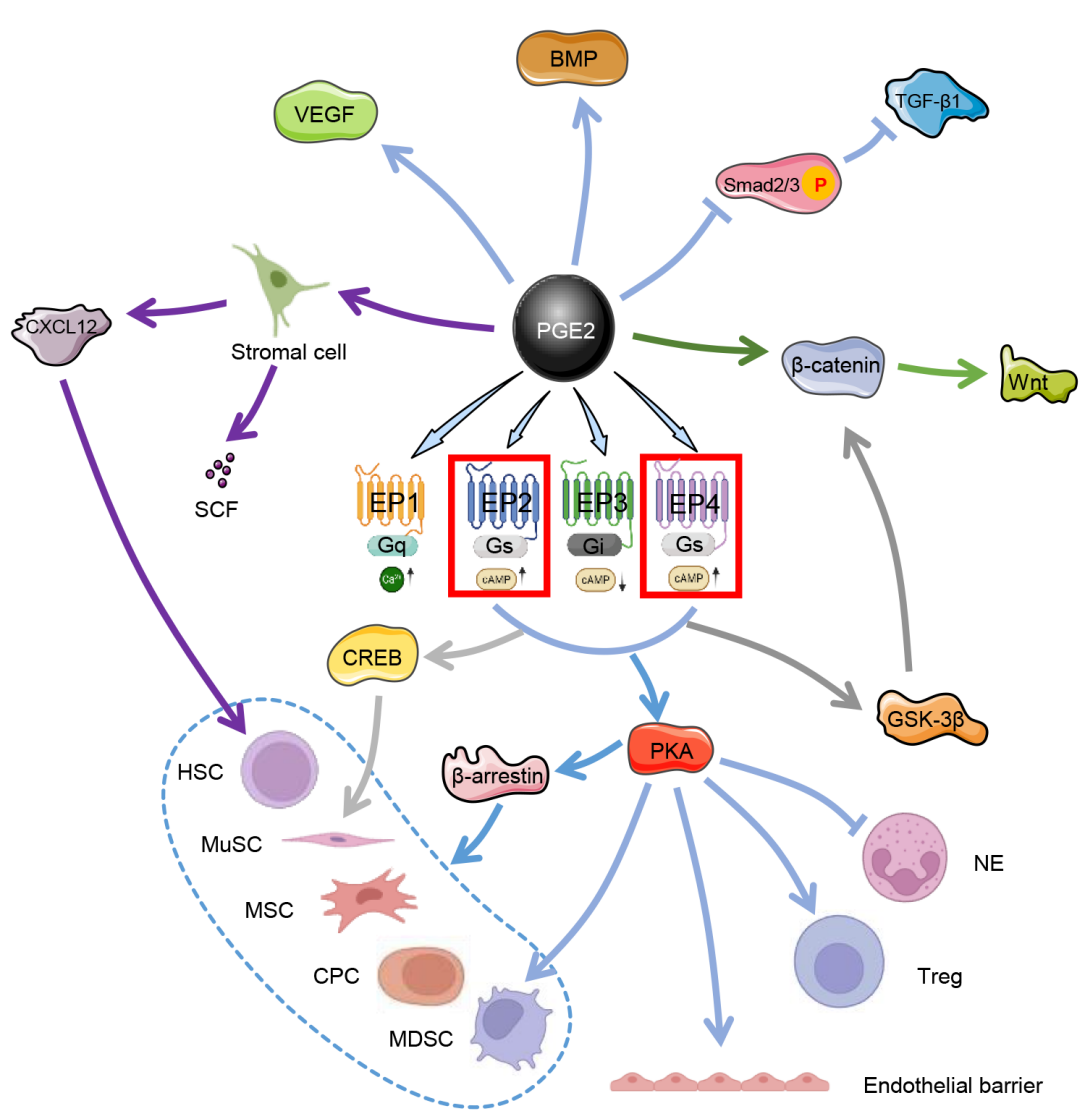
The mechanism of action of PGE₂ in tissue regeneration. PGE₂ orchestrates tissue regeneration by modulating inflammation and immunity via spatiotemporally restricted EP2/EP4-cAMP signaling. It suppresses NF-κB, promotes M2 macrophage polarization, attenuates neutrophil infiltration, and expands Tregs. Concurrently, PGE₂ directly activates stem cells, enhancing expansion, homing, and differentiation, and synergizes with growth factors such as Wnt, VEGF, and BMP to establish a regenerative niche. Its concentration-dependent effects resolve inflammation and promote repair across tissues, with dysregulation contributing to pathology. (Arrows of the same color indicate the same pathway or axis.)

PGE₂ exerts its regenerative influence through multiple conserved mechanisms that are critical for tissue repair across diverse organ systems. These include the modulation of inflammatory responses, the regulation of stem and progenitor cell behavior, and the orchestration of ECM remodeling and angiogenesis (Figure 3(Fig. 3)).

### PGE_2_ acts as a multifaceted regulator of tissue integrity

PGE₂ is a pivotal immunomodulator that orchestrates the critical transition from a pro-inflammatory to a pro-regenerative microenvironment. This is achieved primarily through spatiotemporally constrained EP2/EP4 receptor signaling, which elevates the level of intracellular cAMP to activate PKA (Nazabal et al., 2024[64]). This axis potently suppresses NF-κB-mediated production of pro-inflammatory cytokines, such as IL-6, and drives macrophage polarization toward the pro-resolving M2 phenotype (Gilman and Limesand, 2021[27]). Concurrently, PGE₂ attenuates neutrophil infiltration and expands regulatory T cells (Tregs) (Bai et al., 2018[4]). By tempering the initial inflammatory response, PGE₂ prevents excessive tissue damage and creates a conducive niche for the subsequent activation and function of resident stem and progenitor cells, thereby bridging the gap between initial injury and the onset of tissue reconstitution. Its non-redundant role is evidenced by impaired regeneration following genetic ablation of pathway components or NSAID-mediated COX inhibition (Bryson et al., 2019[7]).

The profound influence of PGE₂ on stem and progenitor cell dynamics is a cornerstone of its regenerative capabilities. This regulation is primarily executed through G protein-coupled EP receptors, with the subsequent activation of the cAMP-PKA signaling axis being a common mechanistic thread (Samiea et al., 2020[77]). In skeletal muscles, PGE₂ binding to the EP4 receptor on muscle stem cells (MuSCs) triggers cAMP-dependent CREB phosphorylation and the induction of the transcription factor Nurr1, a pathway that promotes MuSC proliferation while inhibiting premature differentiation, thereby expanding the pool of cells available for regeneration (Ho et al., 2017[35]). Similarly, in the intestinal crypt, EP4 activation stabilizes β-catenin via the PKA-GSK3β pathway, redirecting stem/progenitor cells toward a wound-associated epithelial fate to expedite mucosal healing (Jackstadt and Sansom, 2017[43]). This fundamental and conserved role underscores its function in dictating stem cell fate decisions across tissues.

PGE₂ plays a multifaceted role in ensuring tissue integrity by coordinately regulating the composition of the ECM and promoting neovascularization. A key anti-fibrotic mechanism involves the suppression of the TGF-β1/SMAD pathway, which inhibits the activation and collagen-producing activity of myofibroblasts, thereby reducing scar formation (Tang et al., 2017[82]). Concurrently, PGE₂ modulates the delicate balance between matrix metalloproteinases (MMPs) and their tissue inhibitors (TIMPs), facilitating controlled ECM degradation and renewal (Altoé et al., 2021[1]). Furthermore, PGE₂ is a potent stimulator of angiogenesis, a process critical in skin wound healing and bone fracture repair. It exhibits concentration-dependent duality, resolving inflammation and accelerating healing in various models, whereas its dysregulation can perpetuate pathology (Chen et al., 2015[10]). By enhancing new blood vessel formation, PGE₂ ensures adequate delivery of oxygen and nutrients to support the metabolic demands of regenerating tissues.

### PGE_2_ orchestrates regeneration in multiple organs

PGE₂ functions as a master regulator of bone metabolism and repair, with its osteogenic effects predominantly mediated through the EP2 and EP4 receptor subtypes. The activation of these Gs-coupled receptors elevates intracellular cAMP levels, which in turn drives the osteogenic differentiation of MSCs, enhances the synthetic activity of mature osteoblasts, and stimulates bone matrix synthesis. The COX-2/PGE₂/EP4 axis is particularly critical during fracture healing, where it supports endochondral ossification and callus maturation (Janssen et al., 2017[45]). Therapeutic strategies, including localized PGE₂ delivery or pharmacological inhibition of its catabolic enzyme 15-PGDH, have been demonstrated to robustly enhance osteogenesis and structural restoration, confirming its status as a promising therapeutic target for bone disorders (Sun et al., 2021[81]).

In skeletal muscle regeneration, PGE₂ acts as a central mediator, primarily through the EP4 receptor on MuSCs. Following injury, locally produced PGE₂ activates EP4, triggering a cAMP-dependent signaling cascade that leads to CREB phosphorylation and the induction of the transcription factor Nurr1 (Yokoyama et al., 2014[93]). This pathway is essential for promoting the expansion of the MuSC pool by stimulating proliferation while actively suppressing premature differentiation, thereby ensuring a sufficient supply of myogenic precursors for effective regeneration (Ho et al., 2017[35]). An age-related decline in this pathway impairs regeneration, a defect that can be reversed by restoring PGE₂ signaling, highlighting its therapeutic potential for sarcopenia.

In addition to bone and muscle, PGE₂ orchestrates repair and regeneration in multiple other organs through sophisticated, context-specific mechanisms. In the skin, it promotes scarless regeneration by enhancing re-epithelialization and angiogenesis while exerting potent anti-fibrotic effects via TGF-β1/SMAD inhibition (Fang et al., 2014[23], Zhao et al., 2016[100]). In the liver, PGE₂ acts as a direct hepatocyte mitogen through EP1/EP4 signaling and modulates Kupffer cell activity to favor a pro-regenerative immune milieu (O'Brien et al., 2014[68]). Cardiac regeneration involves a receptor dichotomy, with EP2 activation driving cardiomyocyte proliferation and EP4 signaling conferring cardioprotection while limiting fibrosis. (FitzSimons et al., 2020[24]). These findings across the skin, liver, and heart underscore the universal role of PGE₂ as a master regulator of tissue homeostasis and regeneration.

Emerging evidence underscores the role of PGE₂ in neural repair processes, primarily through receptor-mediated cytoprotection and modulation of glial activity. While these studies focused on peripheral tissues, analogous mechanisms suggest that PGE₂, via specific EP receptors (notably EP2 and EP4), could promote neuroprotection by mitigating inflammatory damage from activated microglia and astrocytes. The cAMP-PKA pathway, which is central to PGE₂ signaling in other tissues, is a well-established promoter of neuronal survival and axonal growth (Zhao et al., 2025[101]). Furthermore, by polarizing macrophages/microglia toward a regenerative M2 phenotype, PGE₂ may facilitate a permissive environment for axonal regeneration and synaptic remodeling, indicating a promising, although less explored, therapeutic avenue for neurological injuries.

### Key signaling pathways of PGE_2_ to boost regeneration

PGE₂ does not function in isolation but engages in extensive crosstalk with cornerstone developmental and regeneration pathways. A prime example is its synergistic interaction with the Wnt/β-catenin pathway. In intestinal and renal regeneration, PGE₂-EP4-mediated cAMP-PKA signaling inhibits GSK3β, leading to β-catenin stabilization and amplification of Wnt-driven transcriptional programs for stem cell activation and nephrogenesis (Liu et al., 2023[58], Miyoshi et al., 2016[61]). Conversely, PGE₂ antagonizes the pro-fibrotic TGF-β1/SMAD pathway in skin and lung fibroblasts, thereby suppressing excessive collagen deposition and myofibroblast differentiation (Hezam et al., 2023[34]). This intricate dialog with key signaling cascades allows PGE₂ to fine-tune the cellular response to injury, balancing proliferation, differentiation, and matrix remodeling.

**Figure 4:**
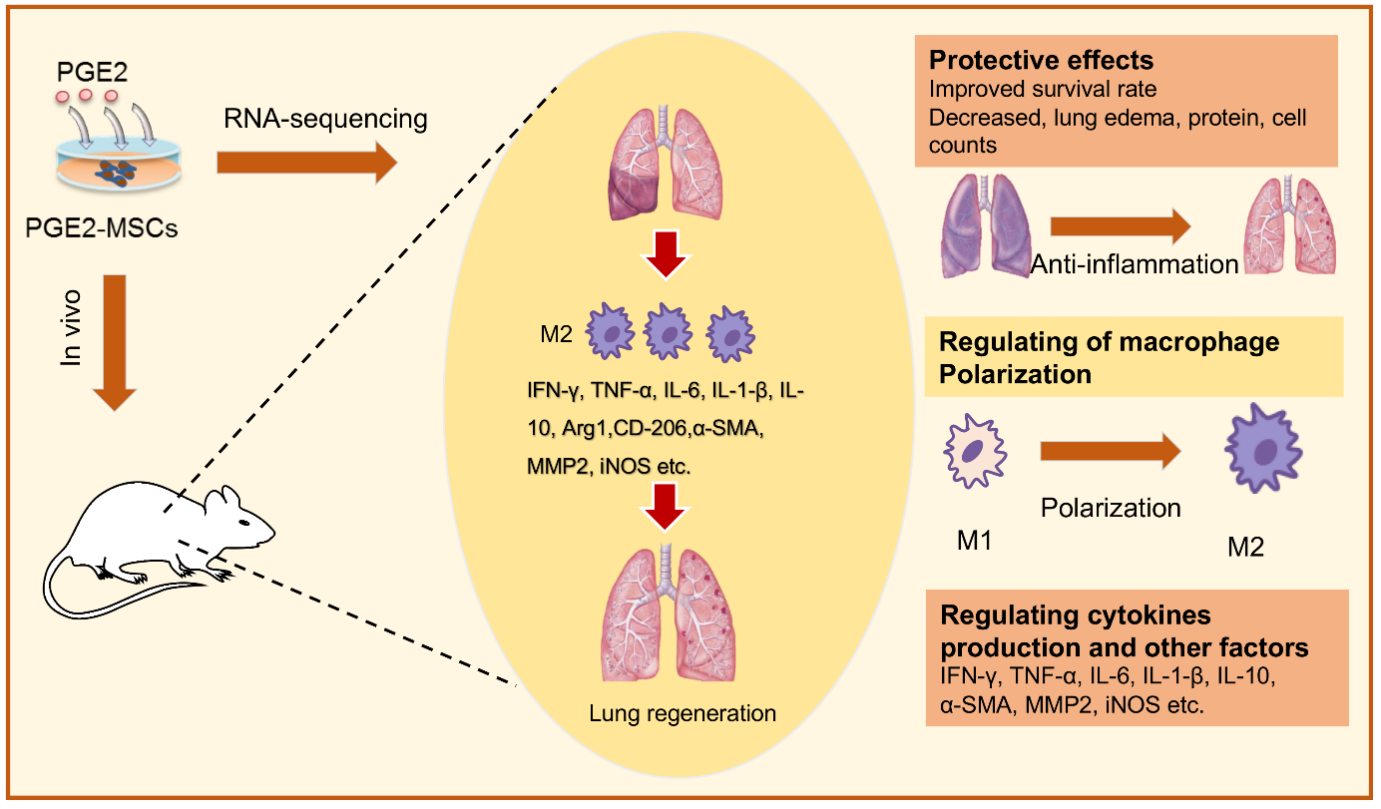
Pretreating MSCs with PGE₂ can ameliorate acute lung injury (ALI) by reducing inflammation and promoting M2 macrophage polarization. Both transcriptomic sequencing and experimental validation indicate that PGE₂ primed MSCs confer protection against ALI by coordinating key processes, including the regulation of macrophage polarization, attenuation of inflammation, enhancement of alveolar fluid clearance, and promotion of tissue regeneration. Reproduced with permission from Ref (Hezam et al., 2023).

PGE₂ exhibits potent synergistic relationships with various growth factors, increasing their reparative potential. This synergy is evident in its ability to prime the tissue microenvironment, for instance, by promoting angiogenesis in concert with VEGF during bone and skin repair. Furthermore, PGE₂ enhances the efficacy of MSC therapies. Ex vivo priming of MSCs with PGE₂ improves their retention, survival, and paracrine activity upon transplantation, leading to superior outcomes in models of lung injury and myocardial infarction. By modulating immune responses and concurrently activating stem cell populations, PGE₂ acts as a force multiplier for growth factor signaling, creating a robust integrated response that is greater than the sum of its individual parts (Figure 4(Fig. 4); Reference in Figure 4: Hezam et al., 2023[34]).

### Epigenetic reprogramming of PGE_2_ pathways balances damage and regeneration

**Figure 5:**
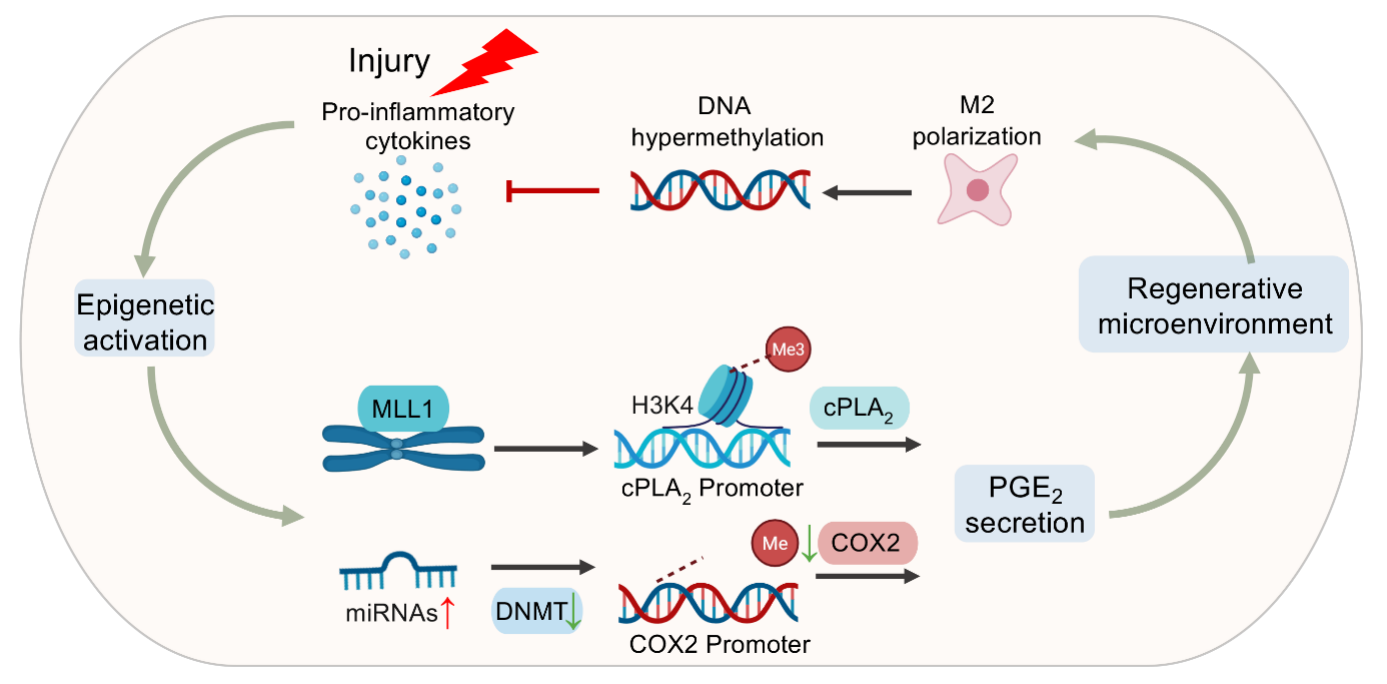
Epigenetic regulation of PGE2 in tissue regeneration. Tissue injury triggers a localized rise in cytokines, which upregulates microRNAs to inhibit the DNA methyltransferases, increase COX-2 expression, promoting PGE₂ secretion. Moreover, an early overexpression of MLL1 in prediabetic wound promotes H3K4 trimethylation at the cPLA₂ promoter, increasing PGE₂ synthesis. Furthermore, PGE₂ contributes to the reconstruction of regenerative microenvironment, promotes M2 macrophage polarization and DNA hypermethylation, inhibiting the injured inflammatory microenvironment.

Epigenetic mechanisms critically orchestrate the biosynthesis and signaling of PGE₂, thereby influencing tissue regeneration outcomes (Figure 5(Fig. 5)). Following tissue injury, histone and DNA modifications regulate key enzymes involved in arachidonic acid metabolism and PGE₂ production. The histone methyltransferase mixed-lineage leukemia-1 (MLL1) catalyzes H3K4 trimethylation at the cPLA₂ promoter, promoting arachidonic acid release for downstream PGE₂ synthesis (Kimball et al., 2017[50]). Moreover, cytokine-induced microRNAs, such as miR-26b-5p and miR-146a-5p, suppress DNA methyltransferases (DNMTs), leading to Cox-2 promoter hypomethylation and transcriptional activation, further amplifying PGE₂ biosynthesis (Durmus et al., 2022[21]). These coordinated epigenetic processes fine-tune PGE₂ levels and ensure the timely transition of macrophages from pro-inflammatory to reparative phenotypes via EP2 receptor signaling, thereby facilitating inflammation resolution and tissue repair. However, excessive or prolonged activation of such epigenetic programs can become maladaptive. For example, pathological overexpression of MLL1 in metabolic or neoplastic conditions results in aberrant chromatin activation and sustained macrophage inflammation, which impairs tissue healing (Tulimilli et al., 2025[83]).

In addition to its regulation by epigenetic enzymes, PGE₂ itself reciprocally modifies the epigenetic landscape in a cell type-specific manner. In macrophages, PGE₂ signaling induces DNA hypomethylation of genes that promote reparative and anti-inflammatory functions, whereas in fibroblasts, it can trigger hypermethylation of anti-fibrotic genes. Through EP2/cAMP activation, PGE₂ enhances DNMT3A expression via the transcription factors Sp1 and Sp3, resulting in global DNA hypermethylation and the silencing of protective genes such as IGFBP2 and MGMT in lung fibroblasts (Huang et al., 2012[40]). Conversely, in fibrotic disorders such as idiopathic pulmonary fibrosis, hypermethylation of the c8orf4 gene suppresses COX-2 expression, diminishing endogenous PGE₂ synthesis and disrupting fibroblast homeostasis (Evans et al., 2016[22]). This dual regulatory feedback mechanism demonstrates that PGE₂ and epigenetic networks jointly establish context-dependent control over regeneration and fibrosis.

Therapeutically, targeting this PGE₂ epigenetic axis has shown promise in restoring physiological repair. Myeloid-specific COX-2 deletion or macrophage-targeted delivery of COX-2 inhibitors via dextran-conjugated nanocarriers reverses pathological epigenetic programming and enhances macrophage reparative polarization in diabetic wounds (Davis et al., 2020[16]). Similarly, DNA methylation inhibitors such as 5-aza-2′-deoxycytidine can restore balanced PGE₂ signaling and fibroblast homeostasis by alleviating pathological hypermethylation (Prasad and Katiyar, 2013[73], Sahin et al., 2013[76]). The integration of COX-2 modulators with epigenetic therapies thus represents a promising strategy to reestablish homeostatic PGE₂ signaling, bridging inflammation resolution and tissue regeneration. However, the outcomes of epigenetic regulation in different damage stages (Kimball et al., 2017[50]) and in pathological wound repair (Davis et al., 2020[16]) can be controversial, and this should be fully considered.

## Translational Opportunities and Challenges for PGE₂-Based Therapies

PGE₂ exerts conserved reparative functions across diverse tissues by coordinating stem/progenitor cell activation, modulating immune responses, and regulating ECM remodeling. Preclinical studies have consistently demonstrated its capacity to accelerate tissue repair in skin, bone, muscle, heart, kidney, and gastrointestinal models. However, clinical translation is limited by rapid enzymatic degradation, dose-dependent biphasic effects, and potential fibrotic or inflammatory complications (Peng et al., 2017[72], Ukon et al., 2022[84]). These limitations highlight the need for advanced therapeutic strategies that can optimize the bioavailability, receptor binding, and spatiotemporal delivery of PGE₂ to safely and effectively realize its regenerative potential.

### The dual role of EP4 in tissue regeneration

The EP4 receptor is a primary mediator of PGE₂ signaling, which originates from the COX pathway. Upon EP4 activation, the Gαs-cAMP-PKA axis is triggered, regulating crucial processes in tissue homeostasis and regeneration (Ukon et al., 2022[84]). In regenerative contexts, PGE₂-EP4 signaling promotes angiogenesis, stem cell differentiation, and ECM remodeling (Wang et al., 2025[87], Zhang et al., 2024[98]). However, EP4 plays a remarkably pleiotropic and context-dependent role across tissues. It exerts anabolic effects in bone, stimulating osteoblast differentiation and minimodeling, while it acts as a catabolic regulator in cartilage, where its inhibition enhances regeneration. Conversely, in the gastrointestinal tract, EP4 activation is cytoprotective and promotes mucosal healing, whereas in the heart, its chronic signaling exacerbates fibrosis through the TGF-β1 and Smad pathways (Kantham et al., 2025[47], North et al., 2010[66], Xu et al., 2024[91]). This tissue-specific duality underscores that the therapeutic exploitation of the axis requires a nuanced understanding of the pathological milieu to harness its regenerative benefits while mitigating adverse effects.

### Interaction of PGE_2_ with stem cells

PGE₂ acts as a pivotal regulator of tissue regeneration. It directly activates tissue-resident stem and progenitor cells and orchestrates synergistic crosstalk with essential growth factor networks (FitzSimons et al., 2020[24], Hsueh et al., 2014[38], Su et al., 2019[80]). By binding primarily to the EP2 and EP4 receptors, PGE₂ initiates conserved intracellular signaling cascades, including the cAMP/PKA and β-arrestin pathways, across diverse stem cell populations. These include HSCs, MDSCs/MuSCs, MSCs, and cardiac progenitor cells (CPCs) (Ding et al., 2019[18], Dong et al., 2020[19], Ikushima et al., 2013[42], Riehl et al., 2020[75]). This signaling significantly augments key stem cell function expansion, self-renewal, homing, survival, and lineage commitment. Mechanistically, PGE₂ drives tissue-specific regenerative programs: it rejuvenates aged muscle stem cells via EP4-cAMP-CREB-mediated epigenetic reprogramming, redirects intestinal stem cells toward a WAE cell fate through the EP4/cAMP/PKA/GSK-3β/β-catenin axis, and promotes the osteogenesis and differentiation of MSCs (Jain et al., 2018[44], Miyoshi et al., 2016[61], Zhang et al., 2002[97]).

Concurrently, PGE₂ functions as a central integrator that amplifies and coordinates key growth factor pathways to establish a regenerative niche. It potently stimulates stromal cells to secrete critical cytokines such as CXCL12 and stem cell factor (SCF), which are essential for HSC maintenance and homing (Desai et al., 2018[17], Smith et al., 2021[79]). PGE₂ exhibits profound synergy with developmental signaling cascades. It stabilizes β-catenin to potentiate Wnt-dependent proliferation in hepatocytes and renal progenitors, cooperating with VEGF to increase angiogenesis, amplifying repair mechanisms in tendons and skeletal muscle, intersecting with BMP signaling to regulate MSC differentiation and suppressing TGF-β1-driven myofibroblast activation by inhibiting Smad2/3 phosphorylation (North et al., 2010[66], Tang et al., 2017[82], Zhao et al., 2016[100]). This dynamic crosstalk establishes PGE₂ as an indispensable amplifier of growth factor efficacy, coordinating cell cycle progression, ECM remodeling, and functional tissue restoration across organ systems. Pharmacological inhibition of its degradation via 15-PGDH blockade further highlights its therapeutic potential by increasing endogenous PGE₂ levels to potentiate these integrated stem cell and growth factor responses.

### Preclinical and clinical trials of PGE_2_

Compelling preclinical evidence has demonstrated that augmenting PGE₂ signaling significantly enhances tissue regeneration across diverse injury models. Pharmacological inhibition of the PGE₂-catabolizing enzyme 15-PGDH or selective agonism of its primary receptors EP2/EP4 accelerates repair in murine models, including >2-fold increased hepatocyte proliferation after hepatic resection, and accelerated hematopoietic recovery by 6 days, with 30-50 % improved platelet/erythrocyte reconstitution after bone marrow transplantation, 30-80 % faster closure with reduced scarring in cutaneous wound healing, diabetic ulcer resolution, renal ischemia-reperfusion injury mitigation, and 70 % reduction in ulceration in patients with colitis (Tang et al., 2017[82], Zhang et al., 2002[97], Zhang et al., 2015[99]). These benefits are mechanistically linked to EP2/EP4-mediated induction of the reparative cytokines CXCL12 and SCF, suppression of the inflammatory mediators, TNF-α, IL-1β and TGF-β1, and enhanced stem/progenitor cell mobilization/function, angiogenesis, and reparative macrophage polarization. Conversely, genetic or pharmacological disruption of PGE₂ synthesis or receptor signaling consistently impairs regeneration, resulting in delayed fracture union, persistent muscle weakness, and compromised cardiac, hepatic, and intestinal regeneration.

Clinically, the therapeutic potential of PGE₂ modulation is demonstrated by the efficacy of ex vivo dimethyl PGE₂ (dmPGE₂)-primed umbilical cord blood HSCs, which increase engraftment in transplant recipients and reduce neutropenia duration (Hagedorn et al., 2014[29]). The established applications of topical PGE₂ analogs include chronic ulcer management, whereas EP4 receptor agonists have exhibited therapeutic efficacy in phase II trials for ulcerative colitis (Kishore et al., 2017[51]). However, clinical translation faces significant challenges: (i) Dose-dependent biphasic effects of PGE₂, which is reparative at low concentrations versus pro-inflammatory at elevated concentrations; (ii) Potential fibrotic or oncogenic risks associated with chronic systemic elevation; (iii) Rapid enzymatic degradation limits bioavailability. These limitations underscore the critical need for advanced spatiotemporal delivery systems, including biomaterial-based hydrogels, platelet-mimetic nanoparticles, and receptor-specific agonists with preferential EP2 over EP4 activation, to optimize the therapeutic index for broad tissue repair applications (Mosa et al., 2008[62], Su et al., 2019[80]).

### Solutions for the translational application of PGE_2_

Preclinical evidence has demonstrated that PGE₂ can enhance osteogenesis, myogenesis, collagen synthesis, mucosal restoration, and fibrosis mitigation in rodent models. Current therapeutic approaches include localized PGE₂ delivery via engineered biomaterials such as injectable hydrogels and nanoparticles, modulation of endogenous biosynthesis through 15-PGDH inhibition via agents such as SW033291, and targeted agonism of pro-regenerative EP2 and EP4 receptors (Chen et al., 2019[11]).

Despite robust preclinical efficacy, the clinical translation of PGE₂-based regenerative therapies remains at an early stage, necessitating accelerated phase I/II trials and focused translational research. Critical barriers include 1) defining human therapeutic windows to balance regenerative benefits against cytotoxic and immunosuppressive risks, 2) developing tissue-specific delivery systems for spatiotemporally controlled bioactivity with minimal off-target effects, 3) establishing long-term safety profiles regarding pro-tumorigenic potential from sustained PGE₂ elevation, 4) resolving discrepancies between preclinical models and human pathophysiology, such as differential EP receptor expression and chronic microenvironments, 5) validating predictive biomarkers, including EP receptor ratios, urinary PGE₂ metabolites, and circulating microRNAs, for patient stratification. Addressing these challenges requires prioritized clinical evaluation of localized delivery platforms and receptor-subtype selective agonists, coupled with scalable good manufacturing practice-compliant production, standardized regenerative end points and collaborative academia-industry-regulatory frameworks.

## Therapeutic Strategies of PGE₂

PGE_2_ orchestrates tissue regeneration by synchronizing EP2/EP4-dependent immunomodulation and stem cell activation. Therapeutic strategies for PGE_2_-mediated regeneration focus on EP4 receptor agonism and 15-PGDH inhibition to increase bioavailability. Despite therapeutic validation via 15-PGDH inhibition in preclinical models, its clinical application requires overcoming context-specific inflammatory risks and stability limitations through precision-targeted delivery approaches**.**

### Pharmacological modulation

On the basis of comprehensive pharmacological evidence, PGE₂ has emerged as a pivotal mediator of tissue regeneration across diverse organ systems. Its therapeutic potential is harnessed primarily through precise receptor-specific modulation, with selective agonism of the EP4 receptor representing a cornerstone strategy (Aringer et al., 2021[3]). EP4 activation orchestrates critical regenerative processes, including VEGF-driven angiogenesis, suppression of fibrosis, and functional restoration in models of myocardial infarction, ulcer healing, and fracture repair (Hatazawa et al., 2007[33], Lee et al., 2000[53], Xu et al., 2025[92]). Conversely, EP4 antagonism impairs regeneration, mimicking the detrimental effects of NSAID-mediated suppression of endogenous PGE₂ synthesis, thereby highlighting the non-redundant role of this receptor (Aringer et al., 2018[2]). The therapeutic landscape in which EP4 is targeted is consequently nuanced. Tissue-specific prodrugs, such as hydroxyapatite-targeted agonists for bone and intestinal alkaline phosphatase-activated formulations for the gut, enable localized delivery, minimizing systemic exposure. While EP4 agonists enhance bone healing and vascular repair, antagonists such as HL-43 and grapiprant show efficacy in mitigating pathological fibrosis, cartilage degeneration, and heterotopic ossification (Chapman et al., 2025[9]). Beyond direct receptor engagement, inhibition of the PGE₂-catabolizing enzyme 15-PGDH potently elevates endogenous PGE₂ levels, accelerating repair in hepatic, renal, and muscular injury models (Chen et al., 2019[11], Desai et al., 2018[17], Liang et al., 2023[56], Wang et al., 2023[86], Zhang et al., 2025[94]). Crucially, the efficacy and safety of PGE₂/EP4 modulation are profoundly context dependent and are dictated by tissue type, disease state, and the inflammatory milieu. Emerging approaches, including biased ligands to decouple the G protein from β-arrestin signaling and combination therapies with biomaterial scaffolds, are being developed to address this complexity. Future success therefore hinges on spatiotemporal control via advanced delivery systems, such as sustained-release hydrogels, to maintain therapeutic concentrations within the regenerative window, thereby maximizing benefits while avoiding context-dependent adverse effects (Table 1(Tab. 1); Reference in Table 1: Blomgren et al., 2020[5]; Huang et al., 2023[41]; Li et al., 2025[55]; Nazabal et al., 2024[64]; Nickel et al., 2016[65]; Sandborn et al., 2023[78]).

### Sustained release of PGE_2_ for the therapeutic application

**Figure 6:**
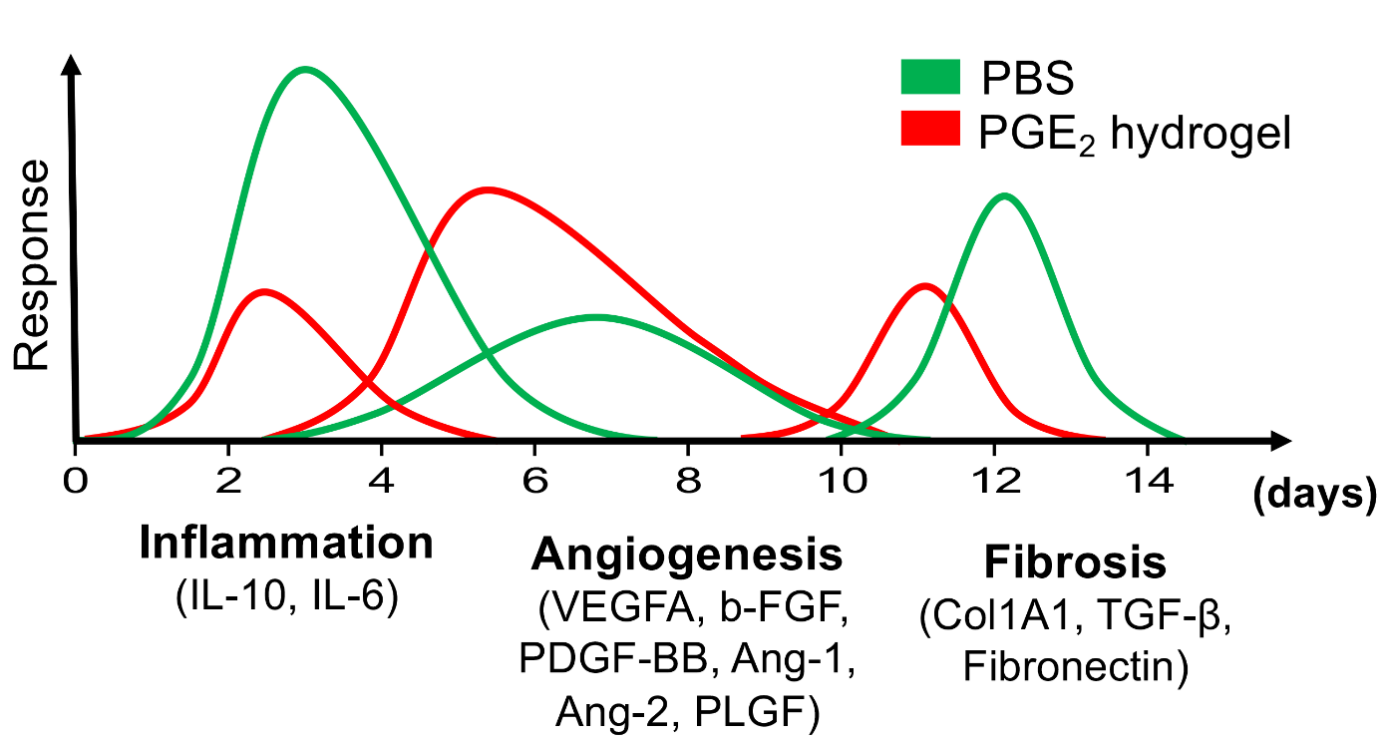
PGE2 can regulate the wound microenvironment and achieve a balance among the three overlapping phases of cutaneous wound healing, inflammation, angiogenesis, and fibrosis, further promoting wound repair. Cutaneous wound healing encompasses distinct yet overlapping phases, including inflammation, tissue regeneration, and remodeling. The PGE₂ hydrogel modulates the wound microenvironment by enhancing macrophage anti-inflammatory and pro-angiogenic activities while concurrently attenuating fibrosis. This integrated therapeutic approach effectively orchestrates balanced progression through the inflammatory, regenerative and remodeling stages, thereby promoting coordinated tissue restoration. Reproduced with permission from Ref (Zhang et al., 2018).

Despite its therapeutic potential of PGE₂, the clinical application of PGE₂ is severely constrained by its rapid inactivation in vivo, primarily through enzymatic degradation by 15-PGDH, resulting in an exceedingly short half-life of approximately 30 s in circulation (Kim et al., 2020[48]). To circumvent this limitation, innovative biomaterial-based strategies focusing on molecular modification and sustained release systems have been developed to increase stability, prolong bioactivity, and improve the delivery efficiency of PGE₂ (Figure 6(Fig. 6); Reference in Figure 6: Zhang et al., 2018[95]).

A highly promising strategy involves the covalent conjugation of PGE₂ to biocompatible polymer matrices. For example, cross-linking PGE₂ to type I collagen via a polyethyleneimine-hydrazinobenzoic acid (PEI-HBA) linker, which forms a pH-sensitive hydrazone bond, has proven effective in achieving controlled and sustained release. The sustained-release PGE₂ matrix exhibited enhanced therapeutic efficacy across multiple disease models. In murine models of AKI, subcapsular implantation of a collagen-PGE₂ matrix promoted functional recovery, attenuated tubular necrosis and fibrosis, and stimulated the proliferation of renal epithelial cells. Mechanistically, PGE₂ was shown to activate the YAP signaling pathway, increasing the number of Sox9⁺ progenitor cells and downstream targets such as amphiregulin (Areg) and survivin, which collectively increase cell survival and regeneration. Similarly, in hindlimb ischemia, the PGE₂ matrix induces robust angiogenesis through the VEGF/VEGFR2 pathway, reduces apoptosis, and activates MyoD1⁺ satellite cells, contributing to muscle regeneration and functional recovery (Huang et al., 2022[39]).

**Figure 7:**
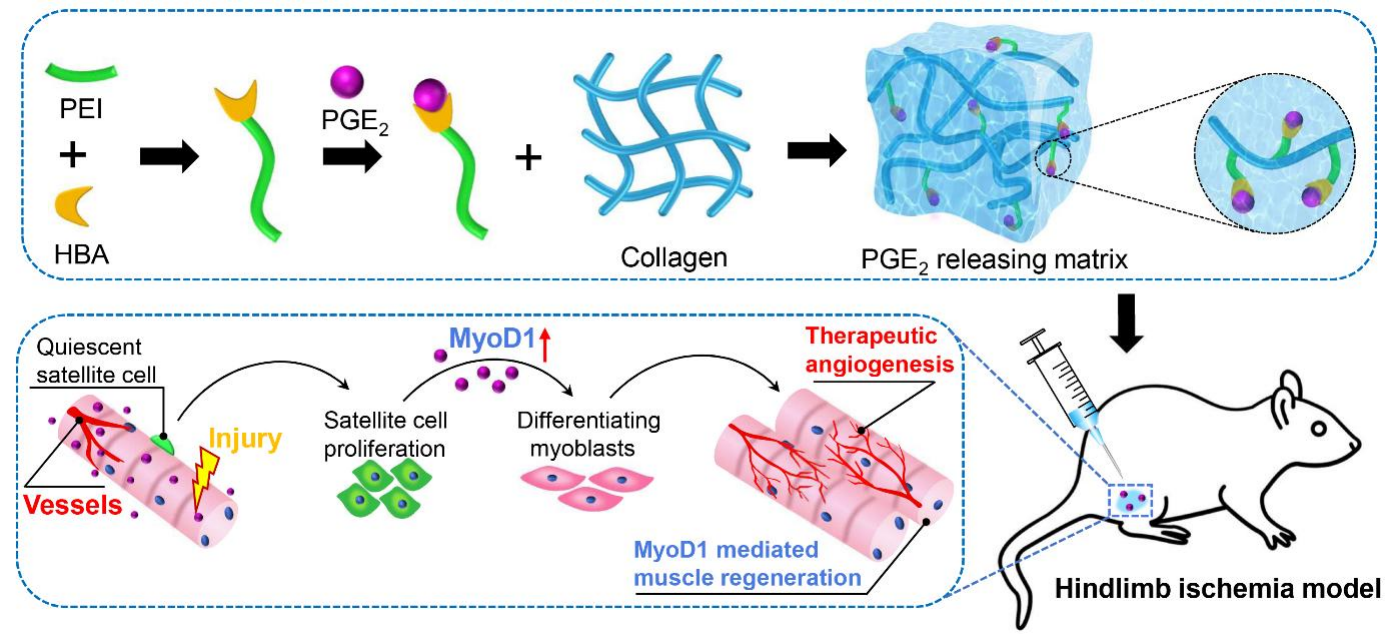
Sustained release of the PGE₂ matrix for therapeutic muscle regeneration. The release of the PGE2 matrix by chemically bonding PGE2 to collagen provides prolonged, localized delivery of PGE₂. This sustained release acts through dual mechanisms; it potently stimulates neovascularization to restore blood flow and directly activates the MyoD1-mediated myogenic program. Together, these coordinated actions promote the repair and regeneration of skeletal muscle, demonstrating significant therapeutic potential for the treatment of hindlimb ischemia. PEI, polyethylenimine; HBA, 4-hydroxybenzoic acid. Reproduced with permission from Ref (Huang et al., 2022).

These engineered matrices mitigate the issue of burst release associated with physical encapsulation methods and provide spatiotemporally controlled delivery, thereby maximizing local bioavailability and minimizing systemic exposure. The combination of biocompatibility, tunable release kinetics, and potent bioactivity makes covalently modified PGE₂ matrices a translatable platform for regenerative medicine. Future efforts should focus on optimizing release profiles, exploring alternative biomaterials, and validating long-term safety and efficacy in large animal models and clinical settings. Such advances will be crucial for harnessing the full therapeutic potential of PGE₂ in a range of ischemic and degenerative diseases (Figure 7(Fig. 7); Reference in Figure 7: Huang et al., 2022[39]).

### Gene therapy and biomaterial-based approaches

Innovative gene therapies and biomaterial platforms are advancing to address the inherent limitations of PGE₂, including its rapid degradation, dose-limiting systemic toxicity, and spatiotemporal specificity requirements. Gene-based strategies enhance localized PGE₂ biosynthesis or receptor signaling. Cardiac-specific COX-2 overexpression reduces infarction in transgenic models, whereas viral vectors delivering COX-2/mPGES-1 or 15-PGDH-targeted shRNA amplify endogenous PGE₂ in stem/progenitor cells (Hoggatt et al., 2013[36], Hoggatt et al., 2009[37], Hsueh et al., 2014[38], Ikushima et al., 2013[42], North et al., 2007[67], Su et al., 2019[80]). Biomaterial systems enable sustained and site-directed delivery, including injectable hydrogels, liposomes and extracellular vesicles that encapsulate PGE₂- or EP4-selective agonists for controlled release. Functionalized scaffolds stimulate endogenous PGE₂ secretion from tissue-resident macrophages, and platelet-membrane-coated nanocells leverage homing mechanisms for infarct-specific targeting (Kim et al., 2008[49], Li et al., 2020[54], Wang et al., 2012[85]). These platforms often integrate PGE₂ with adjunctive agents or preconditioned cells, establishing synergistic regenerative niches that recapitulate endogenous repair dynamics while minimizing off-target effects.

## Emerging Frontiers and Integrative Strategies in PGE₂-Based Regenerative Medicine

Through the activation of the G protein-coupled receptors EP2 and EP4 and subsequent cAMP-PKA signaling, PGE₂ orchestrates a diverse array of pro-regenerative processes. These include the activation and expansion of tissue-resident stem and progenitor cells, the polarization of macrophages toward an anti-inflammatory M2 phenotype, the enhancement of angiogenesis, the suppression of fibrotic pathways via the inhibition of TGF-β1/SMAD3 signaling, and the promotion of epithelial barrier restoration. Preclinical studies have demonstrated promising outcomes using various strategies to augment PGE₂ signaling, such as pharmacological inhibition of its degrading enzyme 15-PGDH, administration of receptor-specific agonists, or biomaterial-assisted local delivery, resulting in accelerated healing in models of bone fracture, muscular injury, gastrointestinal mucosal damage, and cutaneous wounds.

Despite their regenerative potential, the clinical translation of PGE₂-based therapies faces significant challenges. A major limitation stems from its pleiotropic and context-dependent signaling. For example, while EP2/EP4 activation promotes osteogenesis, it can simultaneously inhibit fibroblast migration, which is beneficial for bone repair but detrimental to soft tissue healing. This functional duality, compounded by dose-dependent biphasic effects, undermines therapeutic predictability and target validation. Furthermore, PGE₂ exhibits intrinsic pharmacokinetic instability, with an extremely short half-life necessitating continuous local availability. Conventional delivery systems often fail to achieve spatiotemporally precise release, leading to subtherapeutic concentrations at the injury site, rapid microenvironmental clearance, or systemic diffusion, causing adverse effects such as vasodilation, gastrointestinal disturbances, and potential oncogenicity. Additional concerns include the complexity of EP receptor subtypes. The co-expression of multiple subtypes, which often have opposing functions, complicates therapeutic targeting. Furthermore, the sustained increase in PGE₂, whether achieved by exogenous administration or 15-PGDH inhibition, raises serious safety concerns. These unresolved issues encompass fibrotic responses, cellular senescence, and potential tumorigenic effects in susceptible tissues.

To overcome these challenges, emerging strategies emphasize spatiotemporally controlled delivery and precision targeting. Key advances include the development of subtype-selective receptor modulators. For example, EP4 agonists are utilized to promote angiogenesis and osteogenesis, whereas EP3 antagonists can mitigate fibrotic responses. These approaches are increasingly guided by single-cell receptor expression mapping and patient-specific biomarker stratification, including PTGER gene polymorphisms, COX-2/15-PGDH activity profiles, and epigenetic signatures. In parallel, smart biomaterials such as stimuli-responsive hydrogels and nanoparticles enable microenvironment-triggered release, offering highly localized delivery that minimizes systemic exposure and enhances regenerative efficacy (Bödder et al., 2023[6], Cao et al., 2020[8], Kamolratanakul et al., 2011[46], Zhang et al., 2018[95]).

In addition to unitary applications, PGE₂ serves as an integrative nexus for synergistic regenerative therapies. Its pleiotropic actions make it an ideal candidate for combination strategies that simultaneously target multiple aspects of the repair process. For example, co-delivery of EP4 agonists with osteogenic growth factors such as bone morphogenetic protein-2 (BMP-2) synergistically enhances bone regeneration (Fujimori et al., 2024[25]). Similarly, combining PGE₂ analogs with antifibrotic agents such as pirfenidone potently counteracts pathological extracellular matrix deposition. Cellular therapies have also been augmented through ex vivo priming of MSCs or myeloid-derived suppressor cells with PGE₂, improving their homing capacity, survival, and secretory profile after transplantation. Innovative biomaterial systems, including chitosan/poly-γ-glutamic acid nanoparticles or injectable hydrogels, are being engineered for the co-delivery of PGE₂ modulators with complementary agents, thereby addressing complex regeneration barriers, including chronic inflammation, ischemia, and fibrosis, in a spatially and temporally controlled manner (Mahmood et al., 2021[60]).

In general, harnessing the full therapeutic potential of PGE₂ requires a multifaceted strategy that integrates receptor-specific targeting, smart biomaterial design, and combinatorial treatment regimens. Future efforts should focus on refining spatiotemporal control over PGE₂ signaling, validating subtype-specific agonists and antagonists in disease-relevant models, and developing biomarker-guided approaches for patient stratification. Through these advances, PGE₂-based therapies may soon transition from promising preclinical candidates to effective clinical interventions, ultimately fulfilling their potential as powerful tools in regenerative medicine.

## Conclusions and Future Perspectives

PGE₂ has emerged as a central and evolutionarily conserved regulator of tissue repair, coordinating key processes, including immune resolution, stem cell activation, angiogenesis, and fibrosis suppression, via spatiotemporally controlled signaling through the EP2 and EP4 receptors. While therapeutic strategies such as 15-PGDH inhibition, receptor-specific agonist delivery, and biomaterial-enabled delivery show promising regenerative potential in preclinical models, clinical translation remains limited by metabolic instability, pleiotropic effects, and potential off-target outcomes. Future advances will depend on precision targeting via stimuli-responsive delivery platforms, tissue-selective receptor modulators, combinatorial cue presentation, and biomarker-guided patient stratification. Overcoming these translational barriers will be essential for harnessing the full regenerative capacity of PGE₂ while ensuring its efficacy and safety in clinical applications.

## Notes

Wenlong Wang and Kai Pan contributed equally as first author.

Jun Yang and Zongjin Li (Nankai University School of Medicine,94 Weijin Road, Tianjin 300071, China; E-mail: zongjinli@nankai.edu.cn) contributed equally as corresponding author.

## Declaration

### Conflict of interest

The authors declare no conflict of interest.

### Acknowledgments

This study was financially supported by the National Natural Science Foundation of China (82472169), and the Open funding from the Nankai University Eye Institute (NKYKD202203). Authors thank Andy X. Li from Los Altos, California, USA, for his English editing of this paper.

### Artificial Intelligence (AI) - Assisted Technology

No artificial intelligence was used to conduct this work or prepare this manuscript.

## Figures and Tables

**Table 1:**
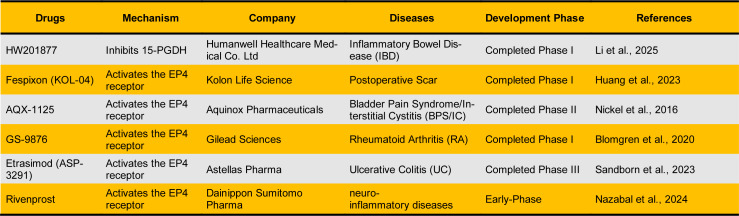
Therapeutic agents that modulate the PGE₂ pathway to promote tissue regeneration

**Figure 1:**
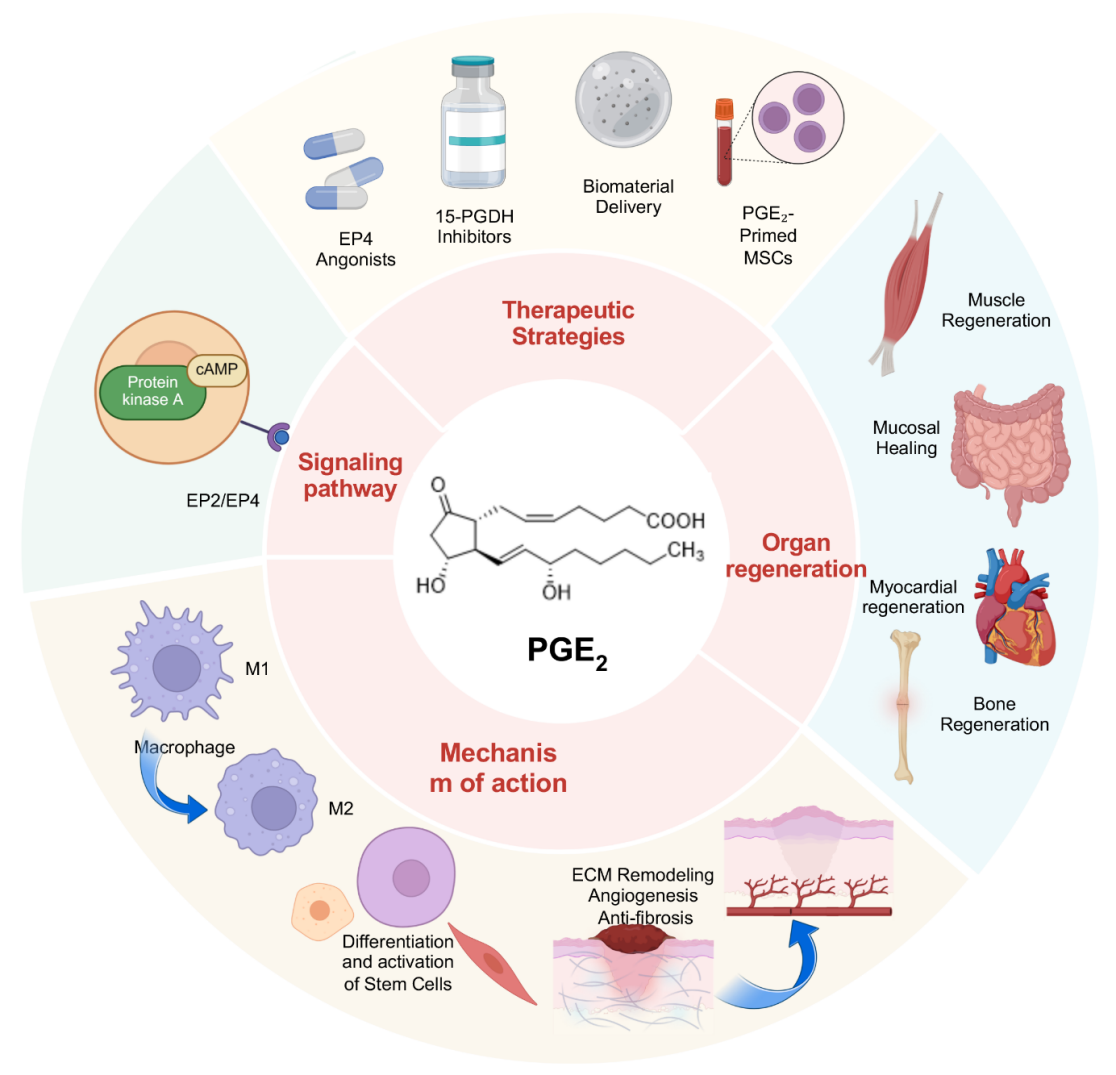
Graphical abstract
